# Evidence of temporal stability in allelic and mitochondrial haplotype diversity in populations of *Glossina fuscipes fuscipes* (Diptera: Glossinidae) in northern Uganda

**DOI:** 10.1186/s13071-016-1522-5

**Published:** 2016-05-03

**Authors:** Robert Opiro, Norah P. Saarman, Richard Echodu, Elizabeth A. Opiyo, Kirstin Dion, Alexis Halyard, Serap Aksoy, Adalgisa Caccone

**Affiliations:** Department of Biology, Faculty of Science, Gulu University, Gulu, Uganda; Department of Ecology and Evolutionary Biology, Yale University, New Haven, CT USA; Yale School of Public Health, Yale University, New Haven, CT USA

**Keywords:** *Glossina fuscipes fuscipes*, Tsetse, Temporal stability, Northern Uganda, Trypanosomiasis, Disease foci, Vector, Effective population size, Bottleneck

## Abstract

**Background:**

*Glossina fuscipes fuscipes* is a tsetse species of high economic importance in Uganda where it is responsible for transmitting animal African trypanosomiasis (AAT) and both the chronic and acute forms of human African trypanosomiasis (HAT). We used genotype data from 17 microsatellites and a mitochondrial DNA marker to assess temporal changes in gene frequency for samples collected between the periods ranging from 2008 to 2014 in nine localities spanning regions known to harbor the two forms of HAT in northern Uganda.

**Results:**

Our findings suggest that the majority of the studied populations in both HAT foci are genetically stable across the time span sampled. Pairwise estimates of differentiation using standardized F_ST_ and Jost’s D_EST_ between time points sampled for each site were generally low and ranged between 0.0019 and 0.1312 for both sets of indices. We observed the highest values of F_ST_ and D_EST_ between time points sampled from Kitgum (KT), Karuma (KR), Moyo (MY) and Pader (PD), and the possible reasons for this are discussed. Effective population size (Ne) estimates using Waple’s temporal method ranged from 103 (95 % CI: 73–138) in Kitgum to 962 (95 % CI: 669–1309) in Oculoi (OC). Additionally, evidence of a bottleneck event was detected in only one population at one time point sampled; Aminakwach (AM-27) from December 2014 (*P* < 0.03889).

**Conclusion:**

Findings suggest general temporal stability of tsetse vectors in foci of both forms of HAT in northern Uganda. Genetic stability and the moderate effective population sizes imply that a re-emergence of vectors from local residual populations missed by control efforts is an important risk. This underscores the need for more sensitive sampling and monitoring to detect residual populations following control activities.

## Background

Tsetse flies (Diptera: Glossinidae) are vectors of human African trypanosomiasis (HAT) and animal African trypanosomiasis (AAT), two diseases that exert a significant constraint on human health, animal production and agricultural livelihood in vast areas of rural sub-Saharan Africa. The occurrence of the two diseases follows the restricted distribution of the tsetse flies across 37 countries, covering more than nine million km^2^ between 14° North and 20° South [1, 2]. In 2000, the African Union recognized trypanosomiasis as “one of Africa’s greatest constraints to socioeconomic development” [3].

HAT is among the most debilitating diseases on the continent, with an estimated 70 million people at risk [4]. The HAT disease presents in two different forms; the Rhodesian form that is restricted to eastern and southern sub-Saharan Africa and is caused by *Trypanosoma brucei rhodesiense,* and the Gambian form that is restricted to central and western Africa and is caused by *T. b. gambiense*. The two trypanosome subspecies are identical morphologically but display stark differences in epidemiological features, and require different diagnosis and treatment methods [5, 6]. AAT, on the other hand, stands as a major obstacle to the development of more efficient and sustainable livestock production systems in tsetse-infested areas [7]. The Programme on African Animal Trypanosomiasis (PAAT) estimates that AAT causes approximately 3 million cattle deaths per year with farmers using approximately 35 million doses of costly trypanocidal drugs [8]. Overall, annual losses in agriculture alone have been estimated at over US $5 billion [9].

In Uganda, about two thirds of the total land area is infested with tsetse flies [10], and this is the only country known to sustain active transmission of both forms of human pathogenic trypanosomes; *T. b. gambiense* in the northwest and *T. b. rhodesiense* in the southeast [11]. There is a growing body of evidence that the disease ranges are expanding [12], and thereby narrowing a disease-free belt of less than 150 km just north of Lake Kyoga [13, 14]. Overlap of the two diseases in north-central districts of Uganda [15] will complicate diagnosis and treatment activities, and provide new challenges, as recombination between the two trypanosome forms can occur in tsetse flies and could lead to novel pathologies [16, 17]. This highlights the need for increased understanding and implementation of vector control and disease prevention measures in this region.

The primary vector of both human and animal forms of trypanosomiasis in Uganda is the tsetse species *Glossina fuscipes fuscipes*, where it is responsible for an estimated 90 % of all disease cases. *Glossina f. fuscipes* belongs to the Palpalis group of tsetse, which inhabits low bushes or forests at the edges of riverine habitats, and like other tsetse species, has a unique reproductive strategy where larvae develop *in utero*. Populations appear to respond to seasonal weather patterns, often disappearing during the bi-annual dry season from sites where they were previously abundant [18], which can cause seasonal bottlenecks and thereby result in large temporal changes in gene frequencies due to genetic drift. However, it has also been hypothesized that larval development *in utero* can stabilize population sizes during dry periods [19], which would result in stable gene frequencies due to low rates of genetic drift. Thus, our understanding of population fluctuations remains incomplete. Likewise, knowledge of tsetse dispersal is also inadequate. Ecological studies using capture-release-recapture data show that tsetse species have a high capacity for dispersal, but genetic data indicate surprisingly high differentiation among populations [20]. Studying temporal patterns of genetic variation can provide insight into some of these knowledge gaps and the apparent contradictions between ecological and genetic data [21]. Temporal population genetics data can also provide estimates of effective population sizes [22], and probability of population bottlenecks, and thereby aid in the choice of spatial and temporal parameters in vector control programmes [23].

Previous work on *G. f. fuscipes* temporal population dynamics has focused on southern, central and southeastern Uganda and has demonstrated stability, with little evidence of seasonal variation in population size [24, 25]. However, regions north of Lake Kyoga have not yet been investigated, and remain a high priority because tsetse from this region are genetically distinct [26–28], and it is where the two forms of HAT will likely merge in the near future [13, 14]. This region also differs significantly in climate, especially in annual precipitation [29]. Southern Uganda has a somewhat cooler climate and is less humid, with mean annual rainfall near Lake Victoria often exceeding 2100–3000 mm; the high temperature varies by 2–3 °C over the year, with a mean daily high being around 26 °C. In the north, the rainfall is between 1000 and 2000 mm, and temperature varies by 5 °C over the year, with the mean daily high being 29 °C [30]. The unique genetic and climatic background of northern Uganda may therefore create different tsetse population dynamics, and thus change the best strategy of vector control for the region.

In this study, we investigate temporal changes in nuclear allele and mitochondrial haplotype frequencies of *G. f. fuscipes* populations in areas north of Lake Kyoga by examining temporal samples collected across multiple locations in both the *T. b. gambiense* and *T. b. rhodesiense* disease belts. We do this by evaluating genetic stability at one mitochondrial marker and 17 microsatellite loci so as to contribute reliable scientific data around which sustainable vector control programs can be planned and implemented.

## Methods

### Tsetse sampling and DNA extraction

**Fig. 1:**
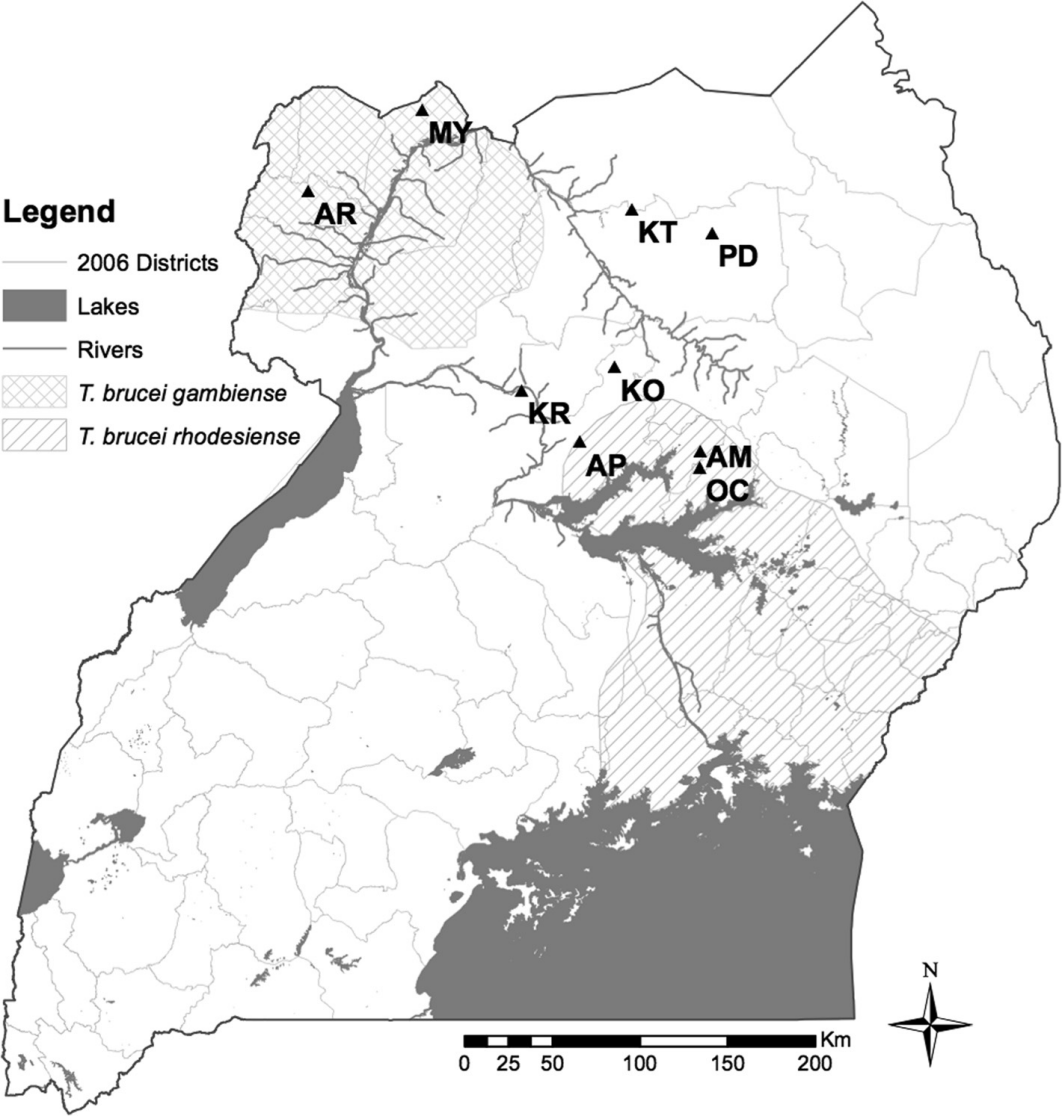
Map of Uganda showing sampling sites. The temporal sites are shown with population codes, as well as the two HAT disease belts in Uganda (*T. b. gambiense* and *Tbr* is *T. b. rhodesiense*)

Tsetse flies were sampled using biconical traps [31] in nine localities spanning two disease foci in northern Uganda during March-December 2014. Populations sampled included Arua (AR), Moyo (MY), Kitgum (KT), Pader (PD), Kole (KO), Apac (AP), Aminakwach (AM), Oculoi (OC) and Karuma (KR) (Fig. 1). We chose sites with genotype data from previous collections done between 2008 and 2011. All captured tsetse flies were preserved individually in cryotubes containing 95 % ethanol. Assuming that *G. f. fuscipes* goes through approximately 8 generations per year [32, 33], our temporal separation ranged from 22 to 52 generations. DNA was extracted from tsetse legs using PrepGEM Insect DNA Extraction kit (ZyGEM New Zealand, 2013), following the manufacturer’s protocols.

### Nuclear microsatellite amplification and genotyping

We evaluated patterns of nuclear DNA (nDNA) genetic diversity using 17 microsatellite loci extensively evaluated for minimal allele dropouts and null alleles and optimized for use in *G. f. fuscipes* in previous studies [24, 25, 27]. Amplifications were performed with fluorescently labeled forward primers (6-FAM, HEX and NED) using a touchdown PCR (10 cycles of annealing at progressively lower temperatures from 60 °C to 51 °C, followed by 35 cycles at 50 °C) in 13.0 μl reaction volumes containing 2.6 μl 5× PCR buffer, 1.1 μl 10 mM dNTPs, 1.1 μl 25 mM MgCl2 and 0.1 μl 5U/μl GoTaq (Promega, USA), 0.1 μl 100X BSA (New England Biolabs, USA), 0.5 μl 10 μM fluorescently-labeled M13 primer, 0.5 μl 10 μM reverse primer, 0.3 μl 2 μM M13-tailed forward primer. For loci C7b and GmL11, 0.5 units of Taq Gold polymerase (Life Technologies, USA) were used instead of Promega GoTaq. PCR products were multiplexed in groups of two or three and genotyped on an ABI 3730xL automated sequencer (Life technologies, USA) at the DNA Analysis Facility on Science Hill at Yale University (http://dna-analysis.yale.edu/). Alleles were scored using the program Genemarker v 2.4.0 (Soft Genetics, State College, PA, USA) with manual editing of the automatically scored peaks.

### Microsatellite marker validation and summary statistics

We evaluated microsatellite genotypes with Genepop v 4.2 [34] to test for deviation from Hardy Weinberg equilibrium (HWE), linkage disequilibrium (LD), and to estimate inbreeding coefficients (F_IS_) using the Markov chain method [35] with 100,000 dememorizations, 1,000 batches and 10,000 iterations per batch. Arlequin v 3.5 [36] was used to calculate observed (H_O_) and expected (H_E_) heterozygosity for each population. Significance values for all multiple tests and comparisons were adjusted using the Benjamini-Hochberg method in preference to the Bonferroni method because of lower incidence of false negatives [37]. We used the program FSTAT v 3.1 [38] to calculate allelic richness and to provide locus and population-specific estimates of microsatellite allele frequencies.

### Mitochondrial DNA amplification, sequencing, and summary statistics

A 570 bp fragment of the region spanning across the mitochondrial DNA (mtDNA) COI and COII gene was PCR-amplified using the primers COII-F1 (5'- CCT CAA CAC TTT TTA GGT TTA G -3') and COI-R1 (5'- GGT TCT CTA ATT TCA TCA AGT A -3') as described by [27] with an initial denaturation step at 95 °C for 5 min, followed by 40 cycles of annealing at 50 °C, and a final extension step at 72 °C for 20 min. We used a reaction volume of 13.0 μl containing 1 μl of template genomic DNA, 2.6 μl 5× PCR buffer, 1.1 μl 10 mM dNTPs, 0.5 μl 10 mM primers, 1.1 μl 25 mM MgCl2, and 0.1 μl (U/μl) GoTaq polymerase (Promega, USA). The PCR products were purified using ExoSAP-IT (Affymetrix Inc., USA) as per the manufacturer’s protocol. Sequencing was carried out for both forward and reverse strands on the ABI 3730xL automated sequencer at the DNA Analysis Facility on Science Hill at Yale University (http://dna-analysis.research.yale.edu/). Sequence chromatograms were inspected by eye and sequences trimmed to remove poor quality data using Geneious v 6.1.8 (Biomatters, New Zealand). The forward and reverse strands were used to create a consensus sequence for each sample, and the sequences trimmed to a length of 404 bp and aligned in Geneious v 6.1.8. DnaSP v 5.10.01 [39] was used to calculate mtDNA haplotype diversity (H_d_) and nucleotide diversity (π).

### Temporal genetic analyses

#### nDNA

We determined the overall proportion of the variance attributable to differences in sampling dates for all 18 sampled points using the analysis of molecular variance (AMOVA) implemented in Arlequin v 3.5; first, we ran AMOVA with two groups containing all sites at generation zero pooled together to form one group and sites with multiple generations forming another. Then, we performed AMOVA with multiple groups by having each temporal sample (generation 0, 22, 27, 29, 35, 38, 48 and 52) forming a separate group.

Genetic differentiation between temporal samples from the same population was quantified by computing pairwise F_ST_ values in Arlequin v 3.5, and Jost’s D_EST_ [40] using DEMEtics [41] in R [42]. It is well documented that high-allelic diversity markers like microsatellites can lead to underestimates of F_ST_ [40, 43, 44]. To account for this potential bias, we standardized F_ST_ values using the formulae developed by Hedricks [43], and used Jost's D_EST_. *P*-values and confidence intervals for Jost’s D_EST_ were also calculated based on 1000 bootstrap resamplings. To test for correlation of differentiation indices and time since first sampling, we ran a linear regression of standardized F_ST_ and D_EST_ against number of generations separating time points sampled in JMP® v11.0 (SAS Institute Inc., Cary, NC, USA, 1989–2007).

#### mtDNA

We employed the same AMOVA strategy outlined above to determine the overall proportion of the variance attributable to differences in sampling dates for the mitochondrial data. We also quantified genetic differentiation between temporal samples from the same population by computing pairwise *Φ*_*ST*_ values between temporal samples in Arlequin *v3.5*, and used the same program to perform Fisher’s exact test of sample differentiation based on haplotype frequencies with 10,000 iterations and 1000 dememorization steps.

### Effective population size (Ne) estimates and tests for bottlenecks

To provide an estimate of effective populations (Ne) of each site, we employed two methods using the nuclear microsatellite markers; the modified temporal method [45] based on [46], and the linkage disequilibrium (LD) method, as implemented in NeEstimator v 2.01 [47]. The temporal method relates the standardized variance of allele frequencies across generations to the effective population size [48, 49] while the LD method uses the level of non-random associations among alleles at different loci [50, 51] to estimate the action of genetic drift, and thus Ne.

Evidence of population bottleneck events was tested using two methods, both of which are implemented in the program BOTTLENECK [52]. The first method tested for an excess of heterozygosity relative to observed allelic diversity [53], and was performed separately for each sample and microsatellite locus. Simulation of heterozygosity at mutation-drift equilibrium distributions assumed the two-phase mutation model (TPM) with 70 % single-step mutations and 30 % of multiple-step mutation, as recommended for microsatellite loci [54], and significance was assessed using Wilcoxon’s signed rank test, as recommended for fewer than 20 loci [52]. The second method tested for a bottleneck-induced mode shift in allele frequency distributions that were based on equal increments of 0.1 [55].

## Results

### Summary statistics

#### nDNA

A total of 404 *G. f. fuscipes* tsetse flies were genotyped using 17 microsatellite loci. Allelic richness ranged from 3.45 in population AM to 4.50 in KR and was generally similar for samples of the same locality analyzed from different time points, except for KR which differed slightly between generation 0 (4.50) and generation 35 (4.08) (Table 1). None of the pairs of loci analyzed exhibited significant LD after correction for multiple testing, which confirmed previous work that these loci are unlinked. However, locus Pg17 exhibited significant departures from Hardy-Weinberg Equilibrium after correction for multiple testing in most of the populations (results not shown), and was therefore dropped from all further analyses. H_E_ and H_O_ values remained almost constant between temporal samples, and showed no evidence of violating Hardy-Weinberg expectations. Site AM (for both generations 0 and 27) had the lowest observed (H_O_) and expected heterozygosity (H_E_) values, while site AR (generations 0 and 52) had the highest H_O_ and H_E_ values (Table 1). None of the F_IS_ values was significantly greater than zero after correction for multiple tests; values were all positive except for one population (OC at generation 0).

**Table 1:** Sample sizes and genetic diversity statistics for 16 nDNA microsatellite loci and an mtDNA marker

			nDNA					mtDNA			
Population	Population code	Date of sampling	N	A_R_	H_O_	H_E_	F_IS_	N	h	H_d_	π
Kitgum	KT-0	January 2012	17	3.88	0.56	0.64	0.1173	9	4	0.6944	0.0021
	KT-22	October 2014	20	3.96	0.59	0.64	0.0792	10	3	0.3778	0.0010
Oculoi	OC-0	July 2011	20	3.51	0.58	0.56	-0.0413	16	6	0.7667	0.0037
	OC-27	December 2014	25	3.56	0.57	0.57	0.0097	10	3	0.5111	0.0014
Aminakwach	AM-0	July 2011	30	3.46	0.52	0.55	0.0507	16	3	0.4917	0.0013
	AM-27	December 2014	25	3.45	0.53	0.55	0.0312	10	3	0.3778	0.0010
Karuma	KR-0	February 2010	60	4.50	0.60	0.68	0.1303	9	3	0.6667	0.0062
	KR-35	July 2014	25	4.08	0.58	0.63	0.0686	10	5	0.8444	0.0085
Apac	AP-0	September 2008	15	3.78	0.50	0.60	0.1668	15	6	0.7619	0.0026
	AP-48	March 2014	29	3.78	0.56	0.59	0.0538	10	5	0.7556	0.0024
Kole	KO-0	July 2010	15	4.14	0.53	0.62	0.1428	10	4	0.7333	0.0025
	KO-29	March 2014	20	4.03	0.61	0.63	0.0282	10	5	0.7556	0.0031
Arua	AR-0	January 2008	15	4.30	0.66	0.67	0.0159	15	3	0.5333	0.0015
	AR-52	June 2014	25	4.28	0.57	0.67	0.1443	10	5	0.6667	0.0025
Pader	PD-0	January 2008	13	4.51	0.63	0.67	0.0905	10	2	0.2000	0.0005
	PD-52	October 2014	15	4.09	0.56	0.61	0.0679	10	4	0.7111	0.0035
Moyo	MY-0	August 2009	15	4.06	0.60	0.65	0.0671	17	3	0.5441	0.0015
	MY-38	June 2014	20	4.01	0.53	0.61	0.1322	10	3	0.5111	0.0014

Sample sizes and genetic diversity statistics for 16 microsatellite loci and an mtDNA marker across nine populations (18 sampled time points) of *G. f. fuscipes* in northern Uganda (Fig. 1). Population codes are shown followed by the time interval (in generations) since the first sampling. *N* number of samples analyzed, *A*_*R*_ allele richness, *H*_*E*_ expected heterozygosity, *Ho* observed heterozygosity, *F*_*IS*_ Fisher’s inbreeding coefficient, *h* number of haplotypes, *H*_*d*_ haplotype diversity, *π* nucleotide diversity. F_IS_ was calculated using Genepop v4.2 [34]; H_O_ and H_E_ were computed in Arlequin v3.5, and A_R_ were estimated using FSTAT v3.1 H_d_, h, and π were computed using DnaSP v5.1

#### mtDNA

For mitochondrial DNA sequences, we recovered a total of 26 haplotypes from the 18 sampled points. Haplotype diversity (H_d_) was moderately high across samples; the number of haplotypes (h) ranged from 2 to 6. Nucleotide diversity (π) was highest in samples from KR (at generations 0 and 35), and lowest in samples from AM (at generation 27) and KT (at generation 22). However, both H_d_ and π remained similar for temporal samples except for KT and PD (Table 1).

### Temporal genetic variation and population stability

**Fig. 2:**
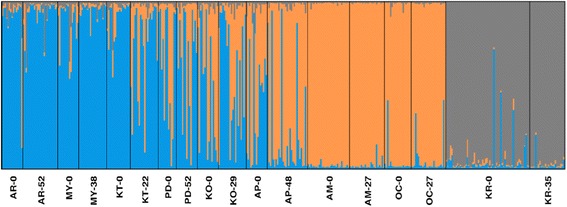
Structure plots of temporal samples. Plots show clustering of samples, organized north to south, with the first time-point sampled followed by the second time-point sampled

Results of AMOVA using both microsatellite and mitochondrial DNA frequencies suggested that differences between samples from different time points do not explain a significant amount of the overall genetic variation. On the other hand, differences among sites contributed significantly to the overall variation (Table 2). STRUCTURE assignment shows that genetic structure remains homogenous from one time point to the next (Fig. 2), and that genetic distance is correlated with spatial distance rather than temporal distance.

**Table 2:** Results of AMOVA testing for temporal genetic structure

	*df*	Sum of squares	Variance components	% variation	*P*-value
nDNA					
2 temporal groups (generation 0 & X)					
Among temporal groups	1	24.368	-0.018	-0.340	0.589
Among sites within groups	16	419.695	0.491	9.370	< 0.0001
Within sites	790	3768.281	4.761	90.97	< 0.0001
8 temporal groups (generation 0, 22, 27, 29, 35, 38, 48, 52)					
temporal groups	7	202.928	0.035	0.66	0.411
Among sites within groups	10	241.135	0.455	8.65	< 0.0001
Within sites	790	3768.281	4.770	90.69	< 0.0001
mtDNA					
2 temporal groups (generation 0 & X)					
Among temporal groups	1	0.255	-0.013	-3.56	0.965
Among sites within groups	16	23.819	0.113	30.59	< 0.0001
Within sites	176	47.673	0.271	72.97	< 0.0001
8 temporal groups (generation 0, 22, 27, 29, 35, 38, 48, 52)					
Among temporal groups	7	6.410	-0.029	-7.94	0.882
Among sites within groups	10	17.664	0.126	34.31	< 0.0001
Within sites	176	47.673	0.271	73.13	< 0.0001

Results of AMOVA testing for temporal genetic structure in nine populations of *G. f. fuscipes* showing the weighted average over 16 microsatellite loci and the same analysis repeated for a mitochondrial DNA marker; degrees of freedom (*df*), sum of squares, variance components, percent (%) variation explained, *P*-value (bold indicates significance at *P* < 0.05). The first analysis for each data type grouped samples at generation 0 and all subsequent generations pooled to form a second group. The second analysis for each data type grouped each generation (0, 22, 27, 29, 35, 38, 48, 52) into separate groups. All computations were done in Arlequin v3.5

#### nDNA

**Fig. 3:**
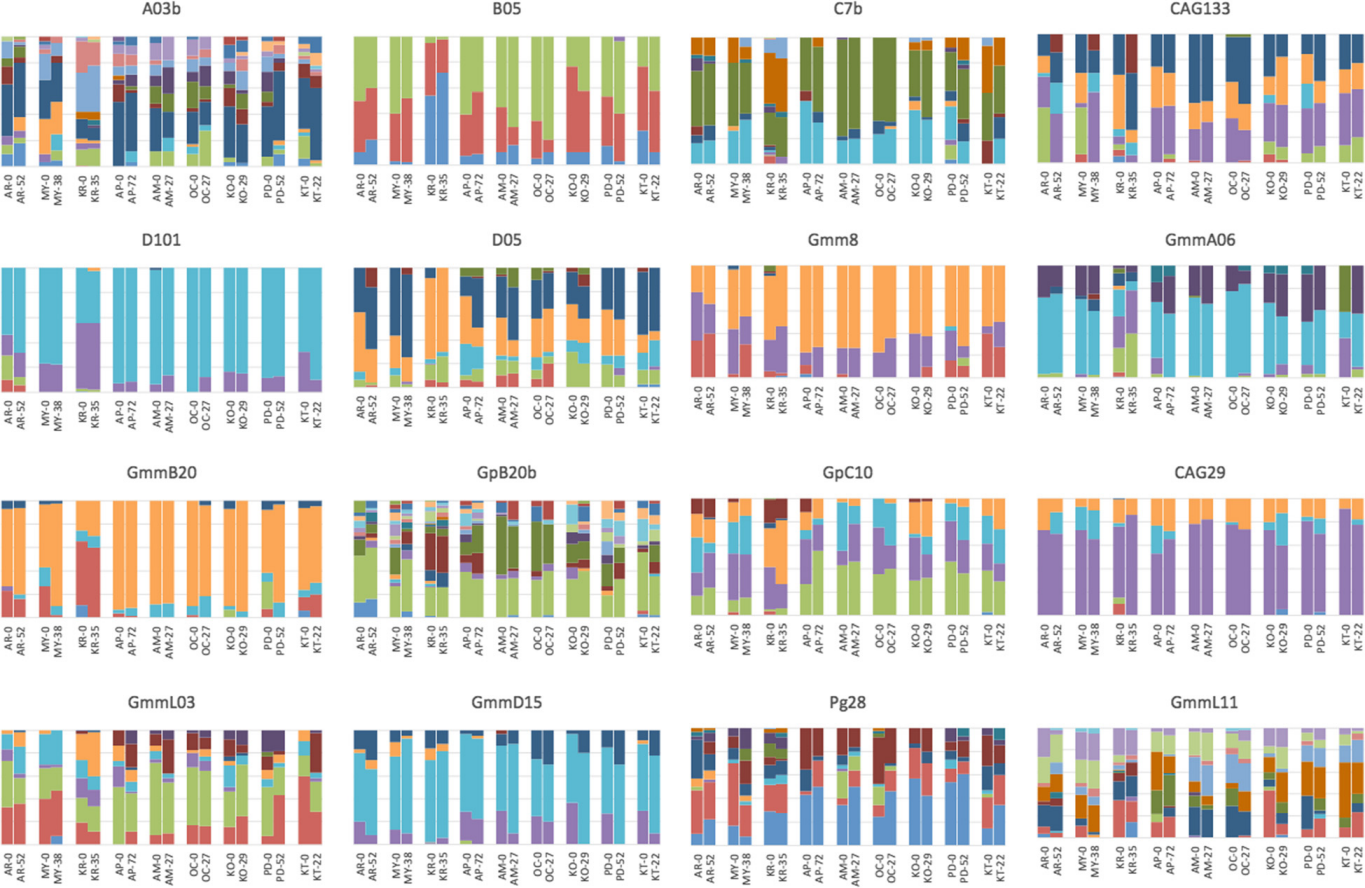
Microsatellite allele frequencies from two time points in nine populations of *G. f. fuscipes* in northern Uganda. Numbers after location code indicate the time interval (in generations) since the first sampling. Vertical bars represent allele frequencies, and each color represents a single allele. The heading of each chart lists the microsatellite locus (A03b, B05, C7b, D101, D05, GmmL03, GmmD15, Gmm8, GpB20b, GmmA06, GmmB20, GmmL11, GpC10, CAG29, Pg28, CAG133)

**Fig. 4:**
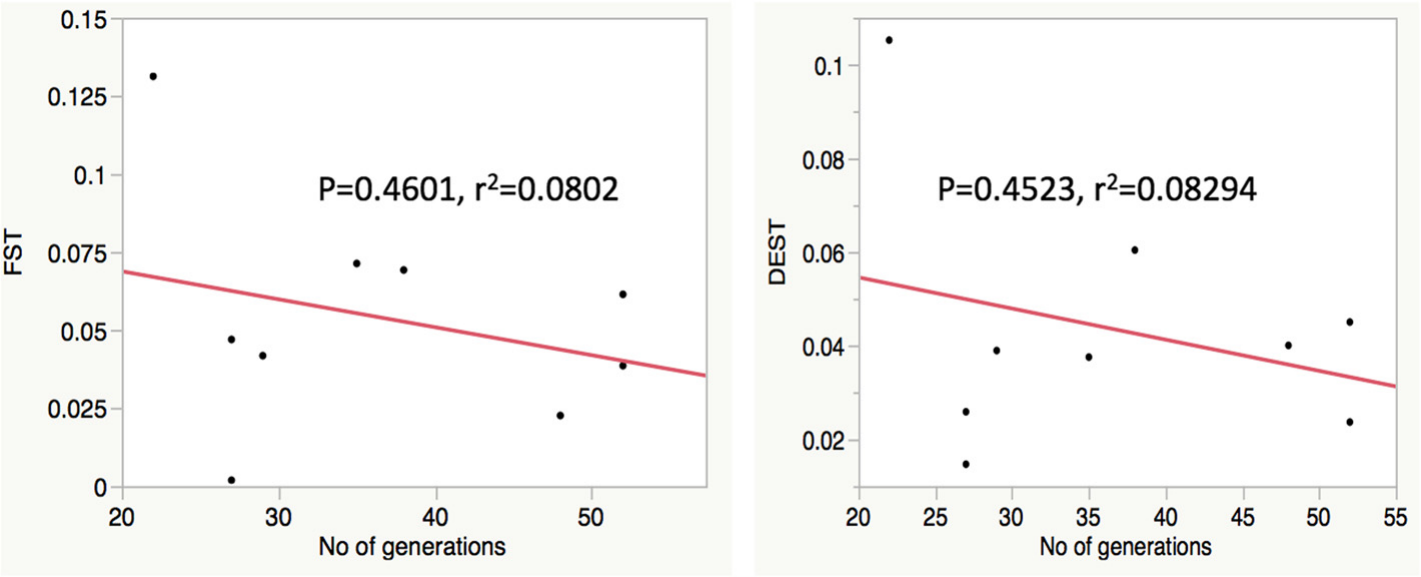
Correlation of genetic differentiation with time between sampling efforts. Linear regression of standardized pairwise F_ST_ (F’_ST_) and D_EST_ against time interval (generations) since the first sampling

Overall, the allele frequency changes were minimal for most of the sites and appear homogenous among temporal samples from the same locations (Fig. 3). Standardized pairwise F_ST_ (F’_ST_) for microsatellites between time points from the same site were generally low (Table 3), with the highest values found between time points at KT (0.0354; *P* < 0.000); MY (0.0692; *P* = 0.001), PD (0.0614; *P* = 0.015) and KR (0.0713; *P* < 0.0001). The moderately high values of F’_ST_ estimates were in concordance with D_EST_ estimates. Number of generations separating sampled time points was not clearly correlated with differentiation indices (*R*^*2*^ = 0.1416, *P =* 0.318; Fig. 4) suggesting that time between our samplings was sufficient to make accurate estimates. As an example, the two temporal populations from AR (AR-0 and AR-52), which were separated by 52 generations, had F’_ST_ and D_EST_ of 0.0385 (*P* = 0.016) and 0.0236 (*P* = 0.003) respectively, whereas other populations separated by fewer generations had similar or even higher values of differentiation indices to AR (Table 3).

**Table 3:** Pairwise estimates of genetic differentiation between temporal samples

		nDNA				mtDNA		
Population	Distance (km)	F_ST_	F_ST_ *P*-value	D_EST_	D_EST_ *P*-value	*Φ*_ST_	*Φ*_ST_ *P*-value	Fisher's test *P*-value
KT-0 *vs* KT-22	36.70	0.1312	0.000	0.1052	0.001	-0.0350	0.991	0.461
OC-0 *vs* OC-27	0.14	0.0019	0.333	0.0147	0.009	0.0237	0.018	0.068
AM-0 *vs* AM-27	0.08	0.0469	0.068	0.0258	0.003	-0.0519	0.658	0.478
KR-0 *vs* KR-35	1.22	0.0713	0.000	0.0375	0.001	-0.0149	0.000	0.624
AP-0 *vs* AP-48	18.87	0.0225	0.171	0.0400	0.005	0.0150	0.181	0.954
KO-0 *vs* KO-29	12.03	0.0417	0.056	0.0389	0.021	-0.0776	0.000	1.000
AR-0 *vs* AR-52	0.87	0.0385	0.016	0.0236	0.003	0.0385	0.703	0.229
PD-0 *vs* PD-52	51.32	0.0614	0.015	0.0450	0.008	-0.0376	0.342	0.179
MY-0 *vs* MY-38	5.86	0.0692	0.001	0.0604	0.003	0.0472	0.000	0.225

Pairwise estimates of genetic differentiation between two temporal samples taken from nine populations of *G. f. fuscipes*. Distance (km) refers to the exact distance in kilometers between trap locations for temporal samples for generation 0 and generation x, microsatellite estimates include F_ST_ and D_EST_, mtDNA marker estimates include *Φ*_ST_ and Fisher’s exact test. F_ST_, *Φ*_ST_ and Fisher's exact tests were calculated in Arlequin *v3.5* while D_EST_ was estimated using DEMEtics in R as the arithmetic mean of the D_EST_ across loci

#### mtDNA

**Fig. 5:**
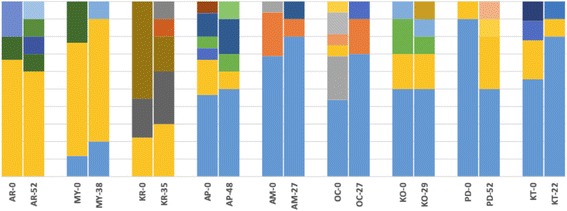
Mitochondrial haplotype frequencies from two time points in nine populations of *G. f. fuscipes* in northern Uganda. Numbers after location codes indicate the time interval (in generations) since the first sampling. Vertical bars represent haplotype frequencies, and each color represents a haplotype. Generally, frequencies of the most common haplotypes remained similar across time points

The same trend was apparent for the mitochondrial DNA haplotype frequencies; some sampled localities gained and others lost haplotypes across time points (Fig. 5). More critically, though, the frequencies of the most common haplotypes remained fairly homogenous for pairs of samples from the same site. The *Φ*_ST_ estimates between time points from the same site were generally low, with the highest values found between time points at MY (0.04715; *P* < 0.0001) and KR (0.01494; *P* < 0.0001).

### Effective population size (Ne) estimates and tests for bottlenecks

Estimates of effective population size (Ne) using the temporal method were all bound by a 95 % confidence interval and ranged from 103 (95 % CI: 73–138) in KT to 962 (95 % CI: 669–1309) in OC. Based on the linkage disequilibrium method, only two populations (AM-27 and KR-35) exhibited 95 % confidence intervals that excluded infinity. Ne estimates using the two methods generally agreed for most populations except for KR, OC and AP. Ne estimates for both KR and OC using the LD method were much lower than from the temporal method (five and eight times respectively). In contrast, Ne estimate for AP using the LD method was higher than the temporal method, and indistinguishable from infinity. For both methods, Ne estimates were not proportional to the number of generations separating temporal samples; for example, OC had the highest Ne estimate using the temporal method at 962 and was based on samples separated by 22 generations, while MY had a Ne estimate close to the overall mean at 313 (95 % CI: 227–412) and was based on samples separated by 38 generations (Table 4).

**Table 4:** Effective population size estimates and tests for population bottleneck events

	Effective population size				Bottleneck		
Population	Ne (Jorde/Ryman)	95 % CI	Ne (LD)	95 % CI	Mean H_E_	TPM *P*-value	Mode shift
KT-0	–	–	Infinite	78.6-Infinte	0.6240	0.470	Normal L-shaped
KT-22	103	73–138	315.4	114.5-Infinite	0.6332	0.161	Normal L-shaped
OC-0	–	–	101.6	36.4-Infinite	0.5564	**0.042**	Normal L-shaped
OC-27	962	669–1309	171.9	91.8–789.2	0.5722	0.137	Normal L-shaped
AM-0	–	–	197.7	68.4-Infinite	0.5445	0.149	Normal L-shaped
AM-27	448	306–615	356.5	142.8-Infinite	0.5477	0.008**	**Shifted mode**
KR-0	–	–	62.3	52.5–75.4	0.6806	0.666	Normal L-shaped
KR-35	468	359–590	83.0	72.2–96.7	0.6274	0.316	Normal L-shaped
AP-0	–	–	Infinite	43.7-Infinite	0.5901	0.609	Normal L-shaped
AP-48	691	491–924	Infinite	536.8-Infinite	0.5898	0.137	Normal L-shaped
KO-0	–	–	252.9	43.3-Infinte	0.6324	0.202	Normal L-shaped
KO-29	363	258–485	382.7	122.0-Infinite	0.6250	**0.039**	Normal L-shaped
AR-0	–	–	Infinite	80.8-Infinite	0.6825	0.217	Normal L-shaped
AR-52	816	593–1074	1379.8	209.7-infinite	0.6663	0.248	Normal L-shaped
PD-0	–	–	51	23.5-Infinite	0.6671	0.372	Normal L-shaped
PD-52	759	551–998	527.6	116.4-infinite	0.6134	0.510	Normal L-shaped
MY-0	–	–	2504.2	55.2-Infinite	0.6380	0.410	Normal L-shaped
MY-38	313	227–412	349.0	129.5-Infinite	0.6126	0.353	Normal L-shaped

Population codes are shown and the number of generations since first sampling, effective population size estimates (Ne) and 95 % confidence intervals (CI), and mean H_E_ as well as the probability value from a bottleneck test. Ne was calculated using two methods; the moments based temporal method of Waples [45] based on Jorde and Ryman [46], and the Linkage Disequilibrium model (LD). Significance of tests for population bottlenecks assumed the two-phase mutation model (TPM) and is displayed as a *p*-value based on 1-tailed Wilcoxon's test, bold indicates *P* < 0.05, bold** indicates significance after Benjamini-Hochberg multiple testing correction (*P* < 0.03889)

Following the Benjamini-Hochberg FDR correction, only one site (AM at generation 27) tested positive for bottleneck events at *P <* 0.0389 under the TPM model (Table 4); however, OC at generation 0 and KO at generation 29 were significant at *P <* 0.05 (Table 4). Additionally, tests for recent bottlenecks using the mode-shift indicator approach indicated that allele frequency distributions in all populations except AM-27 (Table 4) approximated the expected normal L-shape, thus showing no loss of rare alleles via drift as might be expected in a population which has undergone a recent bottleneck event.

## Discussion

We carried out an evaluation of changes in gene frequencies at microsatellite loci and at an mtDNA marker to provide insight into the temporal stability of *G. f. fuscipes* populations that harbor the two forms of HAT in northern Uganda. Generally, temporal population genetics studies are valuable in evaluating population stability and persistence, especially under changes in environment or following a perturbation [56]. Tsetse flies are an interesting study system because they exist in highly structured meta-populations that are sensitive to external environmental change, and because they act as vectors of dangerous human and animal diseases. The presence of the two forms of HAT in Uganda north of Lake Kyoga and the narrowing gap between the two disease belts provides added impetus for monitoring population dynamics of tsetse flies in the region. Although previous work on *G.f. fuscipes* from southern and central Uganda indicated population stability [24, 25], there has not been any work in the genetically unique tsetse populations found north of Lake Kyoga. Temporal stability was also demonstrated for other closely related tsetse fly species like *G. pallidipes* in Kenya [21]. One limitation in previous studies has been short time intervals between samples of only 1–2 years. In this study, we sampled at comparatively longer time intervals, and have demonstrated similar stability of *G. f. fuscipes* in northern Uganda, the region where the two forms of HAT will likely merge in the near future.

Estimates of genetic diversity in both nDNA and mtDNA indicate stable populations that are currently under migration-drift equilibrium. Microsatellite results indicate relatively stable molecular diversity indices (A_R_, H_O_ and H_E)_, while MtDNA sequences show stable h, H_d_, and π except for some populations such as PD and KT (Table 2). These findings indicate stable populations that currently might be under migration-drift equilibrium. Generally low F_IS_ estimates indicate mostly random mating within these populations. The pattern of reduced F_IS_ values in the second sample at all sites except MY suggests a general increase in outbreeding in recent years, potentially driven by migration.

Pairwise estimates of genetic divergence and AMOVA results in both the nDNA and the mtDNA analyses indicated temporal stability of sampled populations, with low estimates of genetic differentiation between temporal samples from the same locality. Exceptions included sites KT, MY and PD, which showed moderate differentiation using both standardized F_ST_ and Jost’s D_EST._ The moderate differentiation observed in the temporal sites KT and PD might be partially attributable to the geographic distance between trapping locations. Trapping of the generation 22 samples from KT was done 36.7 km from the original sample. Similarly, trapping of the generation 52 samples from PD was done 51.2 km from the original sample. Tsetse populations can be genetically distinct even at geographic scales of only 4–5 km, for example, as has been observed in *G. pallidipes* [57], *G.palpalis gambiensis* [58], and even in *G. f. fuscipes* in Uganda [27]. Thus the differentiation we observed in KT and PD may indicate micro-geographic structure between sampling sites rather than temporal instability. On the other hand, two sites (MY and KR) represent potential regions of instability. Both F’_ST_ and *Φ*_*ST*_ values for these sites were highly significant (See Table 3). Temporal sampling efforts for these two sites were not geographically highly separated (5.86 km for MY and 1.22 km for KR), and therefore, these may represent the only areas of instability observed in our study.

Microsatellite-based population size estimates were variable and generally associated with large confidence intervals. Our Ne estimates were lower than most estimates from Uganda obtained by [24] but similar to some localities most proximal to our study sites, such as Masindi (MS). However, our estimates were slightly higher than estimates from southern Uganda around the Lake Victoria basin obtained by [25], and similar to estimates obtained by [59] in other riverine species like *G. palpalis palpalis* in West Africa. Relatively large Ne estimates coupled with evidence of low temporal differentiation indicates genetic drift does not strongly impact these populations [60]. Although Ne is generally difficult to estimate and is affected by many possible biases, the temporal method we have used here is suitable because high allelic richness (Table 1) should outweigh the upward bias in temporal method estimates of Ne [61]. Furthermore, the large sampling intervals used decreases the bias caused by overlapping generations and age structure [62]. The apparently low Ne estimated at some sites, such as KT, may be due to micro-geographic population structure rather than to a true shift in allele frequencies by genetic drift through time, and therefore may represent a false signal of low Ne.

Despite the tendency of *G. f. fuscipes* to exist in discrete patches and the potential for seasonal fluctuations in population size presented by the climate of northern Uganda, there is little evidence of genetic bottlenecks in the populations we studied; only population AM at generation 27, sampled in December 2014, showed evidence of a bottleneck. Our finding of limited bottleneck events is congruent with previous studies in southern Uganda [24, 25, 33]. The apparent lack of extensive seasonal variation in abundance supports the hypothesis that larval development *in utero* may help to relieve tsetse from harsh environmental conditions during reproduction, and hence stabilizes populations [19]. Furthermore, the networks of rivers, streams and other semi-permanent water bodies common in this region may drive overall stability. We hypothesize that waterways may be facilitating connectivity and ‘rescue effects’ between populations. *Glossina f. fuscipes* are known to generally disperse along waterways, following riverbeds or the edges of gallery forests, where they are able to survive low humidity conditions during dry seasons. Additionally, tsetse flies in northern Uganda have not been subjected to extensive control measures. A prolonged period of civil unrest in the region led to a disruption of control efforts and a breakdown in social infrastructure [63], and later control activities were decentralized and are currently managed by underfunded district health and veterinary authorities [64]. At the height of the insurgencies, the populace was displaced into Internally Displaced Persons’ (IDPs) camps leaving tsetse populations in natural conditions for approximately two decades. This time frame was also free of major drought [65], which potentially stabilized population sizes and distribution in the region.

In summary, we find temporal stability in gene diversities of most tsetse populations sampled, which lends further credence to the hypothesis that larval development *in utero* helps to stabilize populations during dry periods, and as a result, decreases seasonal variation in tsetse number. Additionally, large populations of pupa, which develop in the ground over a period of weeks, may also help to ensure the continuity of tsetse populations and would contribute to reducing the variance in genetic changes over time [24]. For northern Uganda, population stability is also probably aided by the presence of many year-round rivers and streams, and historical happenstance, which left tsetse populations in natural conditions free from control efforts for the last several decades.

## Conclusions

The findings point to general temporal stability of tsetse vectors in both foci of the two forms of HAT in northern Uganda. Additionally, migration and gene flow is probably important in shaping the observed genetic stability, which suggests that area-wide control strategies are more desirable than localized efforts. Furthermore, genetic stability and moderate effective population sizes suggest there is a significant risk of re-emergence from local residual populations missed by control efforts. This underscores the need for more sensitive sampling techniques to detect residual populations when tsetse densities decrease due to vector control to avoid a population rebound effect when control and monitoring activities are relaxed. Our results, especially the Ne estimates, may also be useful in evaluating the effectiveness of future control efforts, for instance, by estimating reduction in Ne resulting from control.
